# Epidemiology of mental health disorders in the citizens of Tehran: a report from Tehran Cohort Study

**DOI:** 10.1186/s12888-023-04773-1

**Published:** 2023-04-19

**Authors:** Mahboobe Bahrami, Arash Jalali, Aryan Ayati, Akbar Shafiee, Farshid Alaedini, Soheil Saadat, Farzad Masoudkabir, Nazila Shahmansouri, Ahmadali Noorbala

**Affiliations:** 1 Department of Psychiatry, Behavioral Sciences Research Center, School of Medicine, Isfahan University of Medical Sciences, Isfahan, Iran; 2 Tehran Heart Center, Cardiovascular Diseases Research Institute, Tehran University of Medical Sciences, Tehran, Iran; 3 Department of Emergency Medicine, University of California, Irvine, CA USA; 4 Cardiac Primary Prevention Research Center, Cardiovascular Diseases Research Institute, Tehran University of Medical Sciences, Tehran, Iran; 5 Psychosomatic Medicine Research Center, Imam Khomeini Hospital Complex, Tehran University of Medical Sciences, Dr. Gharib St, Keshavarz Blvd, Tehran, 1419733141 Iran

**Keywords:** Mental health disorders, General health questionnaire-28, Epidemiology, Prevalence, Iran

## Abstract

**Background:**

Mental health disorders (MHD) impose a considerable burden on public health systems. With an increasing worldwide trend in urbanization, urban mental health stressors are affecting a larger population. In this study, we evaluated the epidemiology of mental health disorders in the citizens of Tehran using the Tehran Cohort Study (TeCS) data.

**Methods:**

We utilized data from the TeCS recruitment phase. A total of 10,247 permanent residents of Tehran metropolitan (aged 15 years and older) were enrolled in the study from March 2016 to 2019 via systematic random sampling from all 22 districts of Tehran. The participant's demographic, socioeconomic, and medical characteristics were evaluated by conducting comprehensive interviews. The standardized Persian version of the General Health Questionnaire version 28 was utilized to assess the mental status of the patients according to four central mental health disorders.

**Results:**

Almost 37.1% of Tehran residents suffered mental health problems (45.0% of women and 28.0% of men). The greatest incidence of MHDs was seen in the 25–34 and over 75 age groups. The most common mental health disorders were depression (43%) and anxiety (40%), followed by somatization (30%) and social dysfunction (8.1%). Mental health disorders were more frequent in the southeast regions of the city.

**Conclusions:**

Tehran residents have a significantly higher rate of mental health disorders compared to nationwide studies, with an estimated 2.7 million citizens requiring mental health care services. Awareness of mental health disorders and identifying vulnerable groups are crucial in developing mental health care programs by public health authorities.

## Introduction

Mental health disorders (MHDs) have been associated with a considerable portion of the worldwide burden of diseases. As stated by the latest 2019 global burden of diseases (GBD) reports, mental disorders have continued to account for more than 14% of Years Lost due to Disability (YLD) for approximately 30 years [1]. Based on Global Health Data Exchange data in 2019, the total number of individuals with depression was estimated to be more than 280 million worldwide, with anxiety disorders just as prevalent, affecting more than 300 million individuals [2]. Depression was reported to be the second most significant contributor to global disability (5.5% of all years lived with disability in 2019) [2]. Studies have reported that in 2010, poor mental health cost the world economy 2.5 trillion dollars annually due to declining productivity. This annual cost is projected to increase to 6 trillion by 2030 [3].

Increased migration toward urban areas during recent years has been associated with multiple mental health challenges [4]. Current estimations suggest that by the year 2050, the urban population will rise to 66% of the global population [5, 6]. Socio-economic inequalities, insecurities, and air and noise pollutions are a few factors affecting mental health in urban living settings [4]. Substance abuse is another mental health determinant reported to be significantly associated with urban life [4, 7]. These factors are especially prominent in large metropolitan cities.

Iran is a large country located in the Middle East, and with a population of 86.3 million, it is ranked 18th among the world's most populated countries [8]. The country has been no exception to the worldwide urbanization patterns [9]. Based on 2018 UN reports on human development, Iran's urban population has risen from 64.2% in 2000 to 75.9% in 2019 and is projected to reach 85.8% by 2050 [10]. A 2015 mental health survey of the Iranian population reported a prevalence of 23.4% for MHDs, [11] which is higher than the previous 2004 reports at 20.9% [12]. Depressive and anxiety disorders are among the country's top 10 causes of death and disability, according to 2019 GBD reports [1].

Tehran is the largest city and the capital of Iran, inhabiting more than 10% of the Iranian population. As reported by the 2016 census, Tehran has a population of 8.7 million in the city and 15 million in its metropolitan area [13]. The city is also known as the largest city in western Asia and the 21st in the world [14]. Due to vast immigration from many provinces, Tehran is considered a miniature model of the whole country. Comprehensive studies on mental disorders among citizens of Tehran, a major metropolitan city in the region, are lacking. The latest mental health survey of the Tehran population was conducted in 2009, indicating that 34.7% of Tehran residents suffer from mental disorders. It was also estimated that nearly 2 million Tehran citizens needed mental healthcare services [15].

The results of this study can expand our knowledge of mental health disorders in recent years in Tehran. Understanding the trend of changes in mental health problems can be essential in developing public health programs to prevent and treat mental disorders in major cities such as Tehran.

## Methods

### Study design and setting

The Tehran Cohort study (TeCS) is a multidisciplinary prospective longitudinal study evaluating residents of Tehran. The current analysis is based on the data from the recruitment phase performed between March 2016 and March 2019. The protocol of the study has been previously published [16]. In brief, we contacted 10,000 Tehran households using a systematic sampling method to include a final 5000 households in the study. The inclusion criteria were 1) Participants residing in Tehran at the time of the study, 2) At least one person aged 35 or above was living in their household, and 3) being at the age of 15 years or older. Participants were excluded if they 1) were unable to participate in the study, 2) were not a permanent resident of Tehran, 3) had immigrated to Tehran less than a year ago, and 4) had immigrated from Tehran during the study. The included patients were invited for a recruitment visit to Tehran Heart Center. Home visits were conducted for participants unable to attend the recruitment visits. A research nurse helped the participants who had difficulty completing the study questionnaires.

The Ethics Committee of Tehran University of Medical Sciences approved the protocol of this study (IR.TUMS.MEDICINE.REC.1399.074). Necessary information about the project was shared, and written informed consent was obtained from the participants and their legal guardians (in case they were under 18). The study was conducted at Tehran Heart Center, a tertiary cardiovascular hospital affiliated with the Tehran University of Medical Sciences, Tehran, Iran [17].

### Data collection and measurements

Comprehensive questionnaires were designed to collect participant data for demographic characteristics, socio-economic status, and mental health conditions. The standardized Persian version of the General Health Questionnaire-28 (GHQ28) was utilized to assess participants' status on four central mental disorders (depression, anxiety, somatization, and social dysfunction). The frequencies of these disorders were compared between each gender and age group. The questionnaires that were answered incompletely were excluded from the analysis.

The GHQ‑28 is a 28-item self‑reporting questionnaire to assess current MHDs in community settings [18]. The questionnaire has proven reliable and valid according to abundant studies on normal and clinical populations [19–21]. Nourbala et al. also reported the reliability and validity of the standardized Persian version of GHQ-28 with an 84.7% sensitivity, 93.8% specificity, and an overall misclassification of 8.2% [22]. Three scoring methods have been utilized for this questionnaire [18, 23]. This study calculated the questionnaire score based on the Likert (0–1-2–3) system, as this scoring system is recommended for survey studies [24]. Diagnosis of abnormality in each domain requires a score of ≥ 2 out of 7. A score of ≥ 6 out of 28 indicated the possibility of abnormal mental health.

#### Statistical analysis

Qualitative variables were presented as frequency and percentage. Quantitative data were reported as mean and standard deviation (SD). The age and sex-weighted prevalence of MHDs were calculated using the 2016 Tehran census. The comparison of categorical variables between the two groups was performed utilizing the Chi-Square test.

The distribution of MHDs based on the residence location of the participants was demonstrated on the city map. The first three digits of the postal codes were utilized to locate the participants' district of residence. The geographic maps were illustrated by shp2dta and spmap modules in Stata software (Release 14.2, College Station, TX: Stata Corp LP).

## Results

A total of 10,247 residents of Tehran were included in the study, as previously explained [16]. The population's average age was 48.2 (16.41), and 46.5% were men. Patients were divided into seven age groups. The frequencies and percentages of gender and different age groups are demonstrated in Table 1. The age-adjusted prevalence of each mental symptom in the Tehran population is reported in Table 2. The prevalence of all MHDs was estimated at 37.1% in the Tehran population. The age-adjusted prevalence of MHDs was significantly higher in women (45.0%) compared to men (28.0%). The most common psychiatric symptom was depression, reported in 43.0% (95%CI: 40.4, 45.6%) of the population. The least common condition was social dysfunction, detected in 8.1% (95%CI: 6.7, 9.6%) of the population.

**Table 1:** Frequency and percentage of Gender and age groups in the study population

Age group (year)	Women (%)	Men (%)	Total (%)
15–24	488 (4.8)	455 (4.4)	943 (9.2)
25–34	519 (5.1)	489 (4.8)	1008 (9.8)
35–44	1323 (12.9)	1006 (9.8)	2329 (22.7)
45–54	1202 (11.7)	1007 (9.8)	2209 (21.6)
55–64	1102 (10.8)	865 (8.4)	1967 (19.2)
65–74	600 (5.9)	622 (6.1)	1222 (11.9)
75 +	251 (2.4)	318 (3.1)	569 (5.6)
Total	5485 (53.5)	4762 (46.5)	10,247 (100)

**Table 2:** Comparison of the age-adjusted prevalence of different mental health disorders according to the four domains of GHQ-28 by gender

GHQ Dimension	Total (95% CI)	Women (95% CI)	Men (95% CI)
All dimensions, %	37.1 (34.5- 39.6)	45.0 (41.4- 48.6)	28.0 (24.7- 31.6)
Depression, %	43.0 (40.4- 45.6)	48.5 (44.9- 52.2)	36.8 (33.2- 40.5)
Anxiety, %	40.2 (37.6- 42.8)	48.0 (44.4- 51.6)	31.25 (27.8- 34.9)
Somatization, %	30.5 (28.1- 33.0)	40.1 (36.6- 43.7)	19.5 (16.5- 22.6)
Social dysfunction, %	8.1 (6.7- 9.6)	9.2 (7.2- 11.5)	6.8 (5.1- 9.1)

*CI* Confidence interval, *GHQ* General health questionnaire

**Fig. 1:**
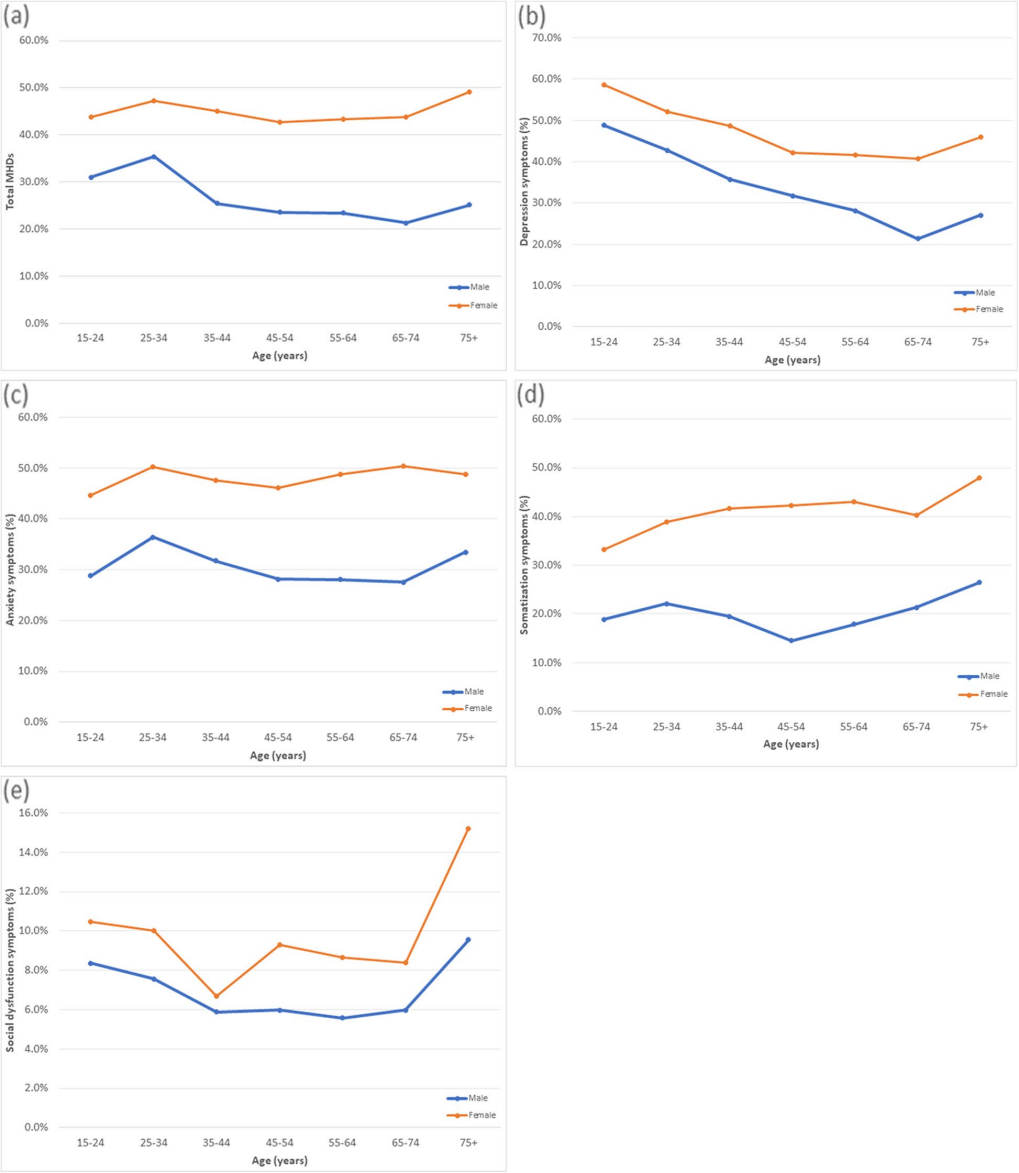
Prevalence of mental health disorders according to four dimensions of GHQ-28 score stratified by gender and age groups. **a**: Total score, **b**: Depression, **c**: Anxiety, **d**: Somatization, **e**: Social dysfunction

Mental health disorders in each domain according to age and sex groups are reported in Fig. 1. Regarding the age groups, the highest rate of MHDs was reported in the 25–34 years and over 75 age group. Notably, the prevalences of all four psychiatric disorders were higher in the female population in all age groups.

The prevalence of depression was the highest in the 15–24 age group (Fig. 1b). This prevalence decreased in the next age groups, followed by an increase in participants aged 75 and older. The lowest rate of depression in both sexes was in the age group 65–74 years. On average, Individuals with depression were four years younger than the rest of the population, revealing an overall decreasing trend for depression by age.

The highest rate of anxiety was seen in the age group 25–34 years, followed by a relatively neutral trend in the following age groups (Fig. 1c). Somatization disorders were highest in subjects aged 75 years and older in both genders. The lowest rates were reported in women aged 15–24 and men aged 45–54 (Fig. 1d). Social dysfunction was most prevalent in subjects over 75 years (Fig. 1e).

**Fig. 2:**
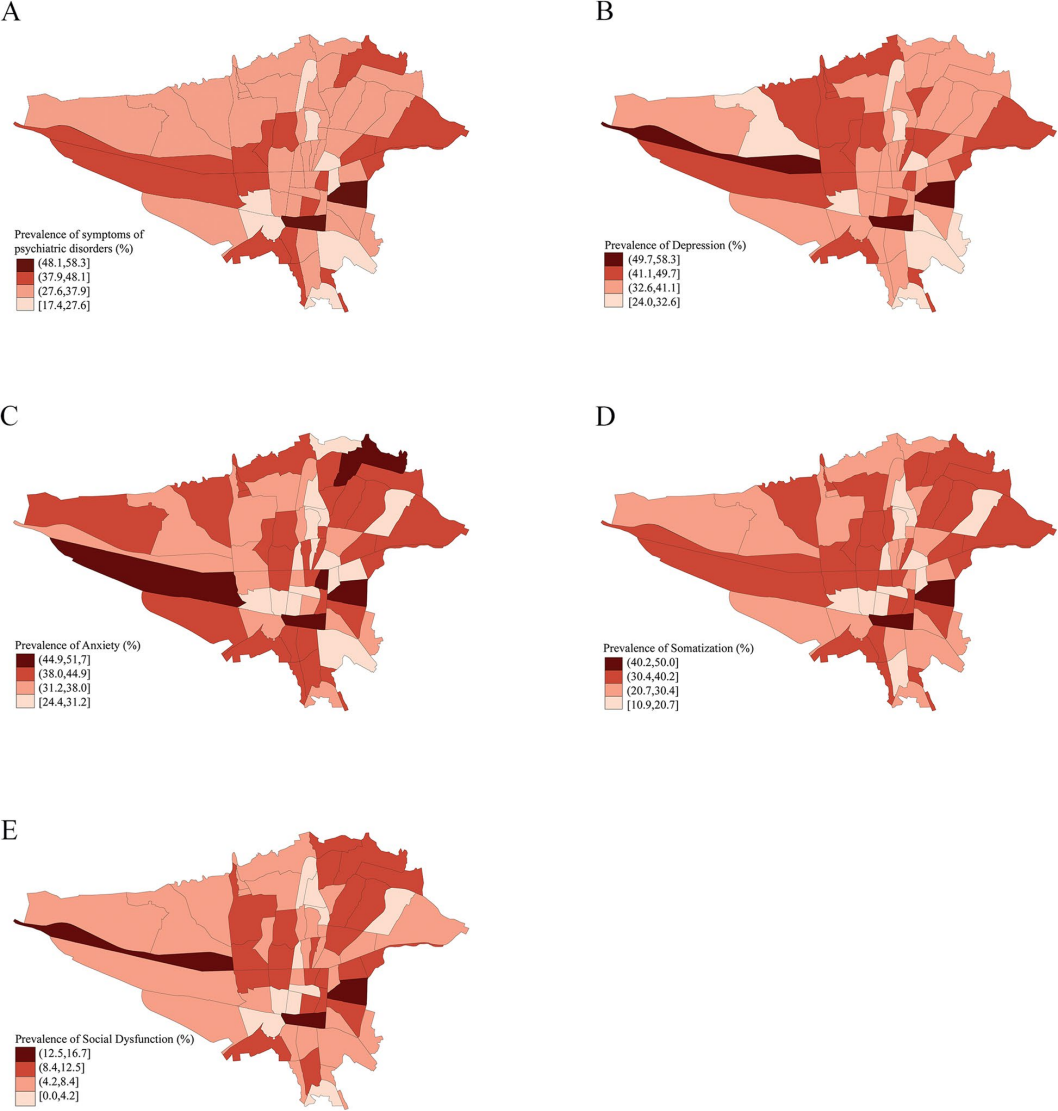
Geographic distribution of mental health symtoms based on GHQ-28 score in postal regions of Tehran. **a**: Total score, **b**: Depression, **c**: Anxiety, **d**: Somatization, **e**: Social dysfunction

The geographic distribution of four dimensions of GHQ mental health disorders based on the participants' zipcodes is illustrated in Fig. 2. Depressive disorders were most commonly reported in the west and southeast of the city. Anxiety disorders were more prevalent in Tehran's west, northeast, and southeast regions. Social dysfunction was most common in the western and southeast areas. Finally, southeast Tehran had the highest prevalence of somatization disorders. Overall, a higher GHQ Score was reported in the southeast of Tehran compared to other regions, indicating a higher prevalence of mental disorders in this region.

## Discussion

The present study aimed to assess the prevalence of mental health disorders in Tehran residents utilizing data from the TeCS with participants from all regions of The city. Four dimensions of mental health disorders were investigated, including depression, anxiety, social dysfunction, and somatization. Based on the GHQ-28 questionnaire, 37.1% of the participants were at risk of mental disorders. This risk was significantly higher in the female residents of Tehran, with a 45% rate. According to these results, nearly 2.7 million Tehran residents are possible cases of MHDs requiring mental health care services, of which 1.6 million are women. These results are concerning and require further attention to its contributing factors and mental health improvement programs.

In a nationwide study by Noorbala et al. in 2015, a significantly lower prevalence of 23.4% was reported for MHDs in the Iranian population [11]. Although a similar assessment tool was used in both studies (GHQ-28), differing results can be due to differences in the sample populations. The greater prevalence of MHDs reported in the population of Tehran compared to the whole country is notable. High exposure to urban mental health stressors (higher living costs [25], pollution [26], traffic [27], inequalities [25], and lack of communication [28, 29]) compared to other regions of the country may be the leading cause of higher MHDs observed in this city [30]. Higher rates of substance abuse compared to other areas can also contribute to the higher MHD prevalence in the city [31, 32]. Nevertheless, additional studies are needed to fully understand the contributing factors to the high MHD prevalence in this city and how to address them.

The previous study on the Tehran population utilizing the same GHQ-28 in 2009 reported a rate of 34.2% for MHDs, lower than the results of the current study [15]. Socio-economic dilemmas, environmental changes, and modern lifestyles may have contributed to the upward trend of MHDs observed in the Tehran population [33–37]. The risk of mental disorders in all main domains was significantly higher in women compared to men in all age groups. These results are consistent with previous studies on worldwide and Iranian populations. Biological factors, sexuality roles, environmental and job stress factors, limitations in sources of satisfaction, and a lack of social participation by women in Iranian society may contribute to the higher prevalence of mental disorders in women than men [11, 38].

According to the results of this study, depression was the most common MHD, followed by anxiety disorders, somatization, and social dysfunction, respectively. Depression and anxiety disorders were reported as the most frequent MHDs stated by World Health Organization and several Iranian surveys [12, 39, 40]. However, in the previous Iranian survey by Noorbala et al., in 2015, anxiety and somatization disorders were reported as the most common MHDs [11]. An increasing trend of depression, especially in the younger population, is reported by many studies around the world [41–43]. The current knowledge of the causes of this increase is limited. However, recent lifestyle changes such as increased obesity [44, 45], decreased sleep [46, 47], higher exposure to media [48], and technological advancements [49, 50] might be contributing to this trend.

Among the various age groups, participants over 75 were observed to have the most MHDs. This finding may result from the fact that older adults with a decrease in physical ability, retirement, the death of a spouse, and loneliness are more likely to suffer from mental illnesses. This result concords with previous Iranian studies reporting increased rates of MHDs in older ages [11, 39].

Contrary to previous studies, the 25–34 age group had the second-highest prevalence of MHDs. Socio-economic and cultural stressors are most prevalent among the 25–34 age group. These individuals are active and productive members of society and have many social, family, and professional responsibilities. The search for a suitable job, financial problems, marriage, substance abuse, and maintaining interpersonal relationships can all contribute to psychological stress. Recent studies also report an increasing trend of MHDs in young adults [51, 52].

Depression was the most prevalent in the 15–24 age group and demonstrated an overall decreasing trend as the age increased. This trend indicates the need for more attention to the mental health of adolescents and young adults.

The lowest prevalence of depression was reported in the 65–74 age group, possibly due to the relative life stability in this age range in opposition to mentioned stressors of young adult life [53, 54]. Another reason may be that depression may manifest as a physical symptom in older ages [55]. The highest prevalence of anxiety was reported in the 25–34 age group, explainable by the stressors mentioned earlier. The prevalence of somatization was highest in subjects aged over 75 years. This could be mainly because individuals in this age group experience a decline in physical strength and often struggle with several chronic diseases. On the other hand, psychological conflicts are more pronounced in the form of physical symptoms at this age.

The highest prevalence of social dysfunction was detected in subjects aged 75 years and higher. Retirement, loneliness, and limited relationships due to physical constraints may contribute to this dysfunction. The calculated frequency of this disorder may be lower than the actual rate, as some individuals with social dysfunction may not have participated in the study due to this disorder.

The southeast region of Tehran reported a significantly higher prevalence of all major MHDs. Various factors may contribute to this discrepancy, including lower socio-economic status, cultural factors, and migration from other cities to this area [56–58]. Previous geographic analyses of poverty and development indices have reported similar patterns in Tehran [59, 60]. Further investigation of these factors and this region's required mental health interventions are necessary.

To the best of our knowledge, this is one of the most extensive studies investigating the prevalence of mental health disorders in all Tehran residents. The results of this study can be essential in developing prevention and treatment programs by identifying populations more vulnerable to MHDs.

### Limitations

The GHQ-28 is a self-reporting tool and cannot replace standard diagnostic tools. Furthermore, this questionnaire can only assess current MHD status and cannot evaluate previous MHDs. Participants who were not included in the study due to the exclusion criteria or lack of response were the study's main limitation. However, the study's large sample size and including participants from all city districts helped overcome this limitation.

## Conclusion

In conclusion, 37% of Tehran residents were suspected of current MHDs, significantly more common in women with a 45% rate. Young adults and the elderly had the most risk of MHDs. According to the study findings, a concerning estimate of 2.7 million Tehran residents requires mental health care services. Depression and anxiety disorders were the most frequently reported MHDs. These alarming results require further attention to the current mental health status of the Tehran population and its underlying causes. Identifying vulnerable groups can be fundamental in developing mental health improvement programs.

## Data Availability

The datasets regarding the current study are available from the corresponding author upon reasonable request.

## Abbreviations

GBD: Global Burden of Diseases
GHQ: General Health Questionnaire
MHD: Mental Health Disorder
SD: Standard Deviation
TeCS: Tehran Cohort Study
UN: United Nations
YLD: Years Lost due to disability
